# Mandatory Mask-Wearing and Hand Hygiene Associated With Decreased Infectious Diseases Among Patients Undergoing Regular Hemodialysis: A Historical-Control Study

**DOI:** 10.3389/fpubh.2021.678738

**Published:** 2021-06-29

**Authors:** Jun-Jian Qin, Yan-Fang Xing, Jian-Hua Ren, Yong-Jian Chen, Ying-Fei Gan, Yan-Qiu Jiang, Jie Chen, Xing Li

**Affiliations:** ^1^Department of Nephrology, The Third Affiliated Hospital of Guangzhou Medical University, Guangzhou, China; ^2^Department of Joint Surgery, The Third Affiliated Hospital of Sun Yat-sen University, Guangzhou, China; ^3^Department of Medical Oncology and Guangdong Key Laboratory of Liver Disease Research, The Third Affiliated Hospital of Sun Yat-sen University, Guangzhou, China; ^4^Shenzhen Ruipuxun Academy for Stem Cell and Regenerative Medicine, Shenzhen, China

**Keywords:** pneumonia, coronavirus disease 2019, hemodialysis, hand hygiene, infectious diseases, mask wearing

## Abstract

**Background:** Infections are the second leading cause of death among patients undergoing hemodialysis. However, preventive measures against infectious diseases are limited and have not been made mandatory for patients.

**Objective:** To investigate the incidence of infectious diseases before and during the coronavirus disease (COVID-19) pandemic.

**Design:** A historical comparative study of a prospective cohort.

**Setting(s):** February 1, 2015 to January 31, 2020 was defined as the period before the mitigative confrontation of the COVID-19 pandemic in China. The period from February 1 to June 29, 2020 was defined as the period of mitigative confrontation of the COVID-19 pandemic in China.

**Participants:** A cohort of patients undergoing hemodialysis whose infectious disease episodes were documented prospectively in the hemodialysis unit of the Third Affiliated Hospital of Guangzhou Medical University since February 1, 2015.

**Methods:** Mandatory mask-wearing and reinforced hand-hygiene education were implemented to prevent COVID-19 from January 23, 2020 in China. The incidence of infectious episodes, including catheter-related infection, digestive tract infection, upper respiratory tract infection (UTRI), pneumonia, and infection at other sites, were documented and compared in the periods before and during the pandemic.

**Results:** The historical control group consisted of 157 patients, with 79 patients in the COVID-19 prevention group. The mask-wearing rate of patients increased from 1.5 to 100%. Hand sanitizer consumption increased significantly during the COVID-19 pandemic. The compliance rates of hand hygiene increased from 66, 75.5, to 55% in physicians, nurses, and other employees before the pandemic to 90.5, 92.5, and 76.5%, respectively. The incidences of UTRI and pneumonia decreased during the pandemic (*p* < 0.001). Notably, catheter-related and digestive tract infections also decreased during the pandemic (*p* = 0.003 and 0.034, respectively). A matched-pair study was conducted to further analyze the 79 individual changes in the incidences of infectious disease before and during the pandemic. As a result, the incidences of UTRI, pneumonia, catheter-related infections, digestive tract infections, and infections at other sites all decreased during the pandemic.

**Conclusions:** The present study indicated an association between mandatory mask-wearing and reinforced hand hygiene education and decreased respiratory, catheter-related, and digestive tract infection episodes in the hemodialysis unit.

## Introduction

Infections are serious complications in patients undergoing hemodialysis. They have been identified as the second leading cause of death and hospitalization for infection in the hemodialysis population (1, 2). A series of measures have been undertaken to reduce the incidence of infections, mainly targeting catheter-related and blood-borne infections (1, 3). However, preventive measures for infections, especially respiratory infections, digestive tract infections, and infections at other sites, were insufficient. Approximately 20% of infections in end-stage renal disease (ESRD) patients are attributable to pneumonia. And evidence suggests that episodes of pneumonia are associated with the particularly high morbidity and mortality in this population. The 30 and 180-day case-fatality rates for pneumonia episodes were 10.7 and 24.8%, respectively (4). The preventive methods against infections were mainly recommendations of hand hygiene, wearing masks when presenting respiratory symptoms, and vaccination, which were based on evidence from the general population rather than from patients undergoing hemodialysis (5, 6). More effective measures are needed to minimize the risk of infection.

The coronavirus disease (COVID-19) has spread globally, and mitigation measures were initiated on January 23, 2020 in China (7, 8). The influence of COVID-19 and its mitigation measures on patients undergoing hemodialysis has been a hot topic since then (5, 9–11). During the pandemic, the COVID-19 mitigation measures were effective for preventing the transmission of the viruses causing infectious respiratory diseases (12–14), which was an unprecedented opportunity to develop cost-effective methods to prevent infections among patients undergoing hemodialysis.

Since February 1, 2015, infectious disease episodes of patients undergoing hemodialysis were documented prospectively in the hemodialysis unit of the Third Affiliated Hospital of Guangzhou Medical University, Guangzhou, China (2). In the present study, we investigated the incidences of infectious diseases before and during the COVID-19 pandemic and evaluated the efficacy of the mitigative measures.

## Methods

### Patient Enrollment

Since February 1, 2015, all patients with ESRD undergoing hemodialysis in the hemodialysis unit of the Third Affiliated Hospital of Guangzhou Medical University were included in a cohort study to document their episodes of infectious diseases with their written consent. Patients with ESRD who were undergoing regular hemodialysis before February 1, 2015 and patients with ESRD initiated on regular hemodialysis between February 1, 2015 and January 31, 2020 were included in the study. The following patients were excluded: patients who underwent hemodialysis for <1 month in the hemodialysis unit, were lost to follow-up within 1 month, tested positive for human immunodeficiency virus, had malignances, were pregnant, or received systemic corticosteroids or immunosuppressive agents. The study was approved by the Clinical Ethics Review Board of the Third Affiliated Hospital of Guangzhou Medical University. Written informed consents were obtained from all the patients at the initiation of hemodialysis.

### Patient Regulation Before and During Mitigative Confrontation to the COVID-19 Pandemic

The period from February 1, 2015 to January 31, 2020 was defined as the period before the mitigative confrontation of the COVID-19 pandemic and that from February 1 to June 29, 2020 was defined as the period of mitigative confrontation of the COVID-19 pandemic in China. Before the mitigative confrontation to the COVID-19 pandemic, the patients and/or caregivers were educated on the prevention of catheter-related infections, such as guidance on bathing with a catheter and the signs of infection. Hand hygiene was suggested for patients with hand sanitizers provided during hemodialysis. Mask-wearing was suggested when patients presented with symptoms of respiratory infections. During the mitigative confrontation of the COVID-19 pandemic, mandatory mask-wearing was enacted for all patients and caregivers. Hand hygiene was strongly suggested and promoted by healthcare workers (HCWs).

### HCWs Regulations Before and During Mitigative Confrontations of the COVID-19 Pandemic

Before the mitigative confrontation of the COVID-19 pandemic, infectious disease prevention strategies were mainly aimed at catheter-related infections. Hand hygiene was routinely recommended. Mask-wearing was mandatory for all HCWs. Hand-hygiene compliance rates of physicians, nurses, and other employees were documented by spot checks every season. Nosocomial infection education and examinations; random inspections of the seven-step handwashing method; germ cultures of washed hands, air, dialysis water, item surfaces of hemodialysis units, and disinfectants; and bacterial endotoxin tests of the dialysis solution and water were conducted every month.

During the mitigative confrontation of the COVID-19 pandemic, the use of disposable surgical caps and masks became mandatory. Nosocomial infection education and examinations were conducted weekly. The frequency of item surface cleaning of the hemodialysis unit increased from one to two times per day before the pandemic to four times per day. Fever and respiratory symptoms among HCWs, patients, and caregivers were routinely monitored. Patients with respiratory infections or fever who were not diagnosed with COVID-19 could continue hemodialysis in the hemodialysis unit.

### Patient Follow-up and Data Collection

Age, sex, causes of ESRD, body mass index (BMI), complications (hypertension, diabetes, cardiovascular disease, cerebrovascular disease, and obstructive nephropathy), hemoglobin, parathyroid hormone, serum calcium, serum phosphorus, and creatinine levels, systolic and diastolic pressures, and, erythropoietin dosage at the time of enrollment were collected. The events related to infectious diseases, such as catheter-related infections, digestive tract infections, upper respiratory tract infections (URTI), pneumonia, and infections at other sites, were prospectively documented during follow-ups. Pathogen data were not included because of a significant amount of missing data. Surveillance videos of patients at the front door of the hemodialysis unit were included to assess the mask-wearing rates before and during the COVID-19 pandemic. For the historical control group under the conventional prevention strategy for infection, the follow-up duration was calculated from February 1, 2015, or the day of hemodialysis initiation to the day of death or that of the last follow-up (ended on January 31, 2020). For the group under the COVID-19 prevention strategy, the follow-up duration was calculated from February 1, 2020 to the day of death or to the day of the last follow-up (ended on June 29, 2020).

### Statistical Analysis

The historical control group consisted of patients with ESRD who were undergoing regular hemodialysis before February 1, 2015 and those for whom regular hemodialysis was initiated between February 1, 2015 and January 31, 2020. The episodes of infection in the historical control group were documented from February 1, 2015 to January 31, 2020. The COVID-19 prevention group consisted of 79 patients receiving regular hemodialysis before February 1, 2020. The episodes of infection in the COVID-19 prevention group were documented from February 1, 2020 to June 29, 2020. In the present study, all hemodialysis patients in the COVID-19 group were evaluated against their paired historical controls. A matched-pair study was conducted to further analyze the 79 individual changes in the incidences of infectious diseases before and during the COVID-19 pandemic.

The Kolmogorov–Smirnov test was used to evaluate the normality of the distribution. Data were reported as median and range for non-normally distributed data. Statistical differences in clinical characteristics between the two groups were compared using the *t*-test or the Mann–Whitney test for continuous variables, Pearson's chi-squared test, or continuity correction for categorical data. Correlations between different parameters were analyzed using Spearman's rank correlation test. Excel 2016 spreadsheet software (Microsoft) was used for data input and collection, and R software (version 3.6.0; R Project for Statistical Computing) and SPSS version 22.0 (IBM) statistical software were used for descriptive and statistical analyses. Statistical significance was defined as a 2-sided *P*-value < 0.05.

## Results

### Patient Characteristics

**Figure 1:**
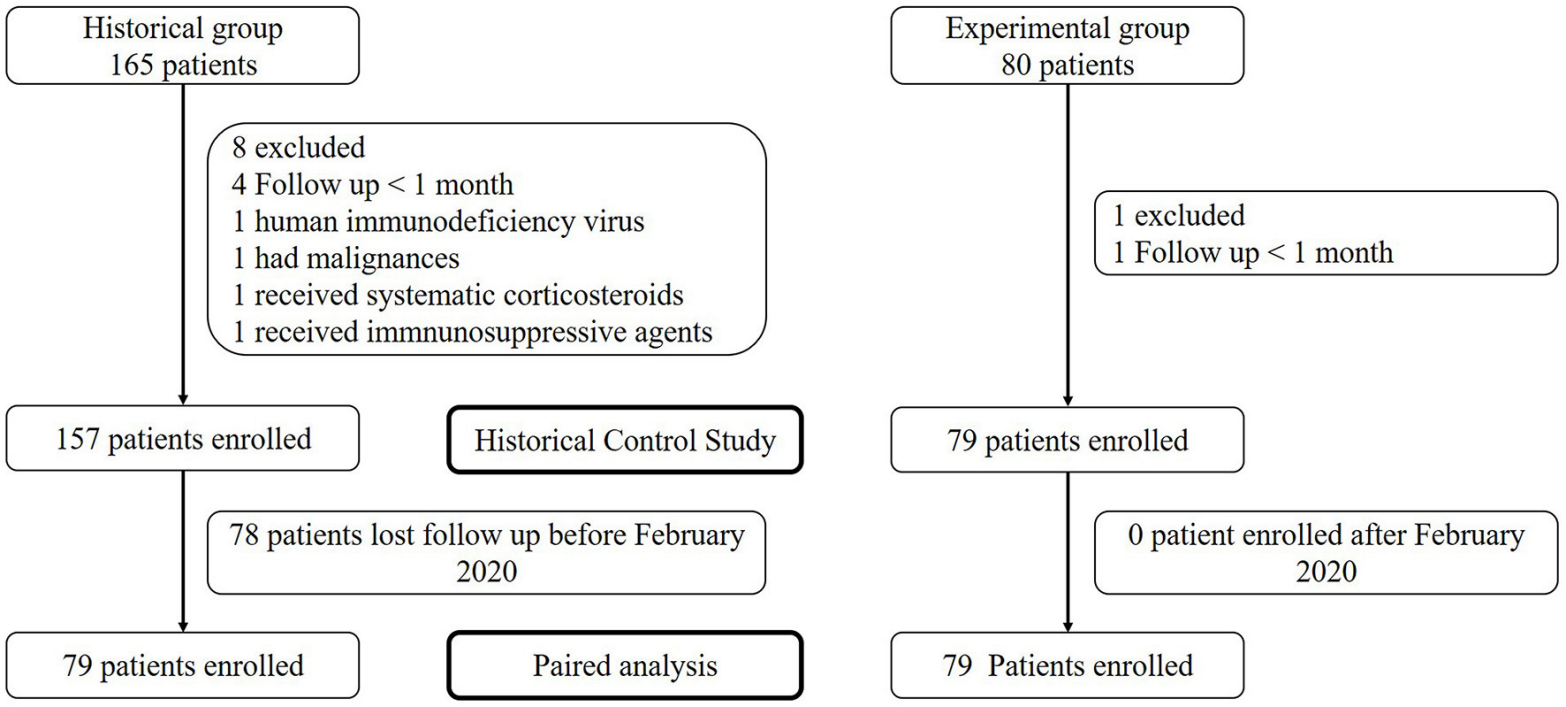
Patient flow diagram.

The historical control group consisted of 157 patients with a median of 39.5 months (range 1–60.8 months) of follow-up, and the COVID-19 prevention group consisted of 79 patients followed-up for 5 months. All 79 patients in the COVID-19 group had corresponding historical controls. Forty-nine (31.2%) patients in the historical control group died before the pandemic. No patient died or was lost to follow-up in the COVID-19 prevention group (Table 1, Figure 1).

**Table 1:** Hemodialysis patient characteristics before and during mitigative confrontation of the COVID-19 pandemic.

	**Before *N* = 157**	**During *N* = 79**	***P*-value**
Age (year, median, range)	62 (24 – 92)	60 (24 – 88)	0.045
Gender (*n*, %)			0.961
Male	84 (53.5)	42 (53.2)	
Female	73 (46.5)	37 (46.8)	
End-stage renal disease cause (*n*, %)		0.681	
Polycystic kidney	6 (3.8)	4 (5.1)	
Hypertension	19 (12.1)	9 (11.4)	
Obstructive nephropathy	7 (4.5)	1 (1.3)	
Diabetes	60 (38.2)	33 (41.8)	
Transplant kidney failure	5 (3.2)	4 (5.1)	
Chronic nephritis syndrome	57 (36.3)	28 (35.4)	
Others	3 (1.9)	0	
Body mass index (median, range)	21.3 (13.3 – 44.4)	21.6 (14.0 – 29.0)	0.821
**Complications (*****n*****, %)**			
Hypertension	151 (96.2)	77 (97.5)	0.722
Diabetes	57 (36.3)	32 (40.5)	0.530
Cardiovascular disease	29 (18.5)	15 (19.0)	0.923
Cerebrovascular disease	38 (24.2)	14 (17.7)	0.257
Obstructive nephropathy	10 (6.4)	3 (3.8)	0.552
Hemoglobin (g/L, mean ± S.D.)	96.8 ± 17.2	101.0 ± 16.6	0.054
Parathyroid hormone (μmol/L, median, range)	436 (10 – 3,000)	534 (10 – 3,000)	0.262
Serum calcium (mmol/L, mean ± S.D.)	2.26 ± 0.21	2.31 ± 0.18	0.136
Serum phosphorus (mmol/L, mean ± S.D.)	2.12 ± 0.64	2.22 ± 0.65	0.289
Creatinine (μmol/L, mean ± S.D.)	965 ± 276	1,046 ± 239	0.027
Systolic pressure (mmHg, median, range)	140 (100 – 180)	145 (112 – 180)	0.152
Diastolic pressure (mmHg, median, range)	80 (57 – 130)	83 (57 – 97)	0.145
Erythropoietin dosage (IU/week) (*n*, %)		0.174	
4,000	27 (17.2)	11 (13.9)	
8,000	57 (36.3)	40 (50.6)	
12,000	73 (46.5)	28 (35.4)	
Death	49 (31.2)	0 (0.0)	0.000
Length of Follow-up (month, median, range)	39.5 (0.9 – 60.8)	5 (5 – 5)	0.000
Length of Hemodialysis (month, median, range)	52 (6 – 191)	59.5 (6 – 191)	0.430

The patients undergoing hemodialysis in the historical control group and those in the COVID-19 prevention group had similar characteristics. The causes of ESRD were diabetes (38.2 and 41.8%) and chronic nephritic syndrome (36.3 and 35.4%, respectively). The complications were hypertension (96.2 and 97.5%), diabetes (36.3 and 40.5%), cardiovascular disease (18.5 and 19.0%), and cerebrovascular disease (24.2 and 17.7%, respectively). The COVID-19 prevention group presented a slightly lower age and higher serum creatine levels (Table 1).

### Adherence to Infection Prevention Regulation in Patients Undergoing Hemodialysis, Caregivers, and HCWs

According to the surveillance videos, the mask-wearing rate of patients increased from 1.5 to 100%. Hand-sanitizer consumption increased significantly during the COVID-19 pandemic, which indicated an increased hand-hygiene rate of patients and HCWs. All the HCWs passed the nosocomial infection-related examinations. The results germ cultures of washed hands, air, dialysis water, item surfaces of hemodialysis units, and disinfectants were all qualified, as well as the bacterial endotoxin test results of dialysis solution and water. The compliance rates of hand hygiene increased from 66% (range 39–93%) in physicians, 75.5% (range 67–86%) in nurses, and 55% (range 34–71%) in other employees before the pandemic to 90.5% (range 87–94%), 92.5% (range 89–96%), and 76.5% (range 72–81%), respectively (Table 2).

**Table 2:** Adherence to infection prevention regulation of patients, caregivers, and HCWs in hemodialysis unit before and during mitigative confrontation of the COVID-19 pandemic.

	**Before *N* = 16 season**	**During *N* = 2 season**	***P*-value**
Hand sanitizer consumption (× 500 ml/season, median range)	11 (10 – 18)	22.5 (21 – 24)	0.013
**Hand hygiene compliance rates of HCWs (%, range)**			
Physicians	66 (39 – 93)	90.5 (87 – 94)	0.052
Nurses	75.5 (67 – 86)	92.5 (89 – 96)	0.013
Other employees	55 (34 – 71)	76.5 (72 – 81)	0.013
Mask-wearing rate of patients (incidence/observed individuals, %)	3/194 (1.5)	239/239 (100.0)	<0.001

*HCWs, healthcare workers*.

### Comparisons of Infectious Diseases Episodes in the Patients Undergoing Hemodialysis Before and During the COVID-19 Pandemic

**Figure 2:**
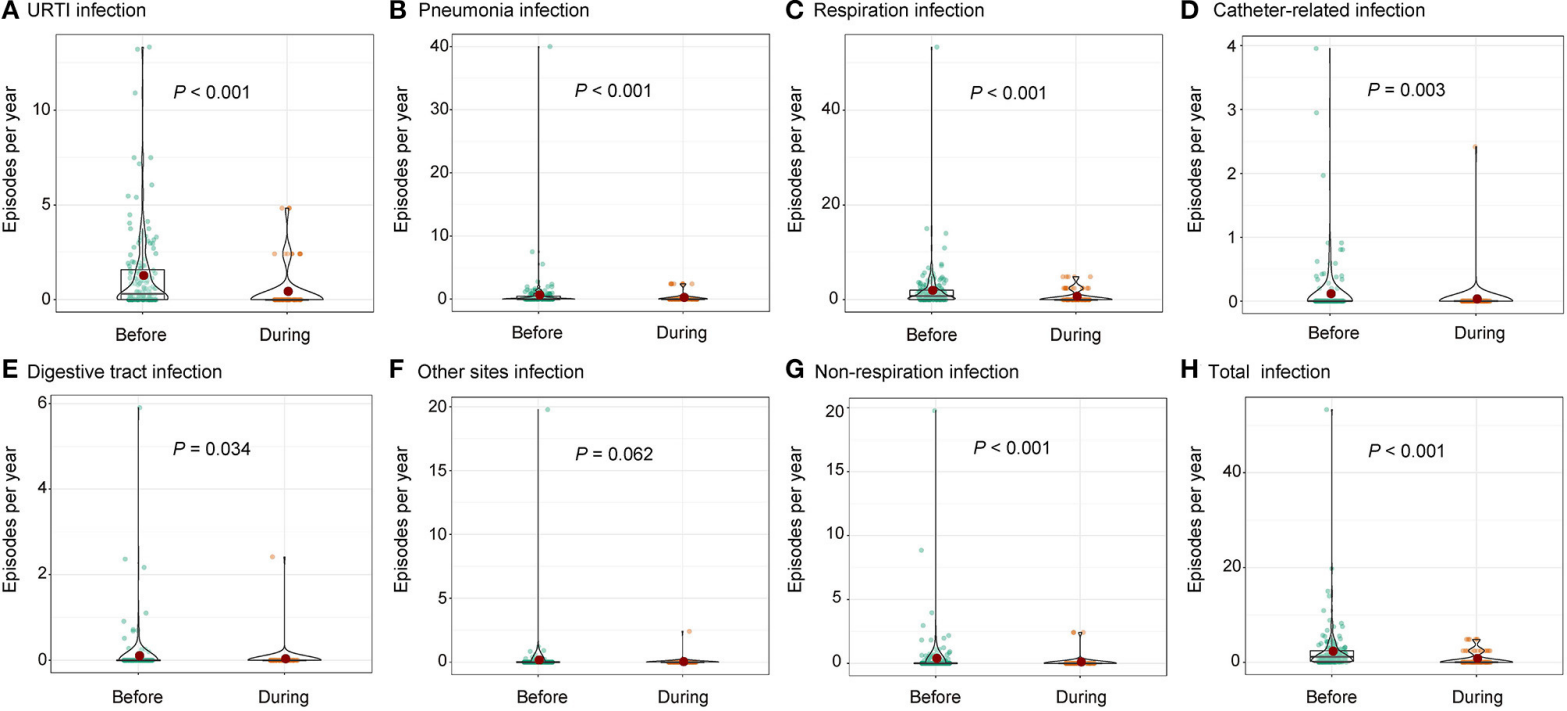
Comparison on infectious episodes before and during the COVID-19 pandemic including **(A)** URTI, **(B)** pneumonia, **(C)** respiration infection, **(D)** catheter-related infection, **(E)** digestive tract infection, **(F)** other sites infection, **(G)** non-respiration infection, and **(H)** total infection. URTI, upper respiratory tract infection.

None of the patients were diagnosed with COVID-19. The mean incidence of URTI and pneumonia decreased from 0.1057 and 0.0538 to 0.0356 and 0.0204 per month per patient, respectively, during the COVID-19 pandemic (*p* < 0.001). Notably, catheter-related and digestive tract infections also decreased by more than 50% during the pandemic (*p* = 0.003 and 0.034, respectively). However, the decreases in infections at other sites, mainly urinary tract infections, were not significant (Figure 2 and Table 3).

**Table 3:** Comparison on infectious episodes before and during the COVID-19 pandemic.

**Episodes per month Mean ± Std. Deviation**	**Before *N* = 157**	**During *N* = 79**	***P*-value**
URTI	0.1057 ± 0.1847	0.0356 ± 0.0954	<0.001
Pneumonia	0.0538 ± 0.2735	0.0204 ± 0.0611	<0.001
Respiratory infection	0.1585 ± 0.3956	0.0561 ± 0.1160	<0.001
Catheter-related infection	0.0093 ± 0.0375	0.0025 ± 0.0226	0.003
Digestive tract infection	0.0085 ± 0.0460	0.0025 ± 0.0226	0.034
Other sites of infection	0.0133 ± 0.1320	0.0025 ± 0.0226	0.062
Total infection	0.1907 ± 0.4140	0.0637 ± 0.1230	<0.001

*URTI, upper respiratory tract infection*.

**Figure 3:**
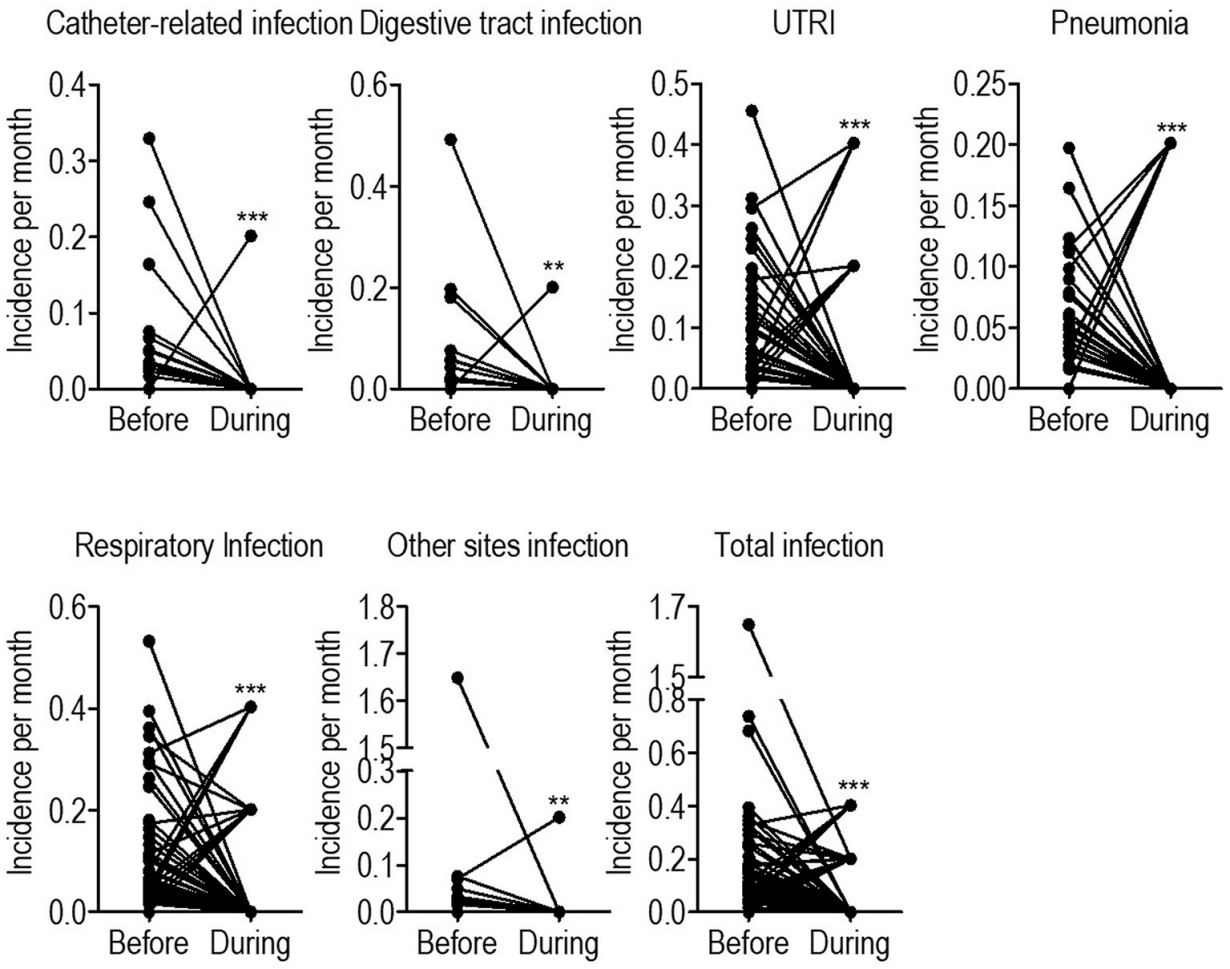
Historical paired comparison on infectious episodes before and during the COVID-19 pandemic. URTI, upper respiratory tract infection. *p < 0.05; **p < 0.01; ****p < 0.001.

A matched-pair study was conducted to further analyze the 79 individual changes in the incidence of infectious diseases before and during the COVID-19 pandemic. As a result, the median incidences of UTRI, pneumonia, catheter-related infections, digestive tract infections, and infection at other sites decreased during the pandemic significantly. Thus, the total infection incidence decreased during the pandemic, according to the matched-pair study (Figure 3 and Table 4).

**Table 4:** Historical paired comparison on infectious episodes before and during the COVID-19 pandemic.

**Episodes per month Mean ± Std. Deviation**	**Before *N* = 157**	**During *N* = 79**	***P*-value**
URTI	0.0595 ± 0.0932	0.0357 ± 0.0954	<0.001
Pneumonia	0.0231 ± 0.0396	0.0204 ± 0.0611	<0.001
Respiratory infection	0.0826 ± 0.1100	0.0561 ± 0.1160	<0.001
Catheter-related infection	0.0164 ± 0.0511	0.0025 ± 0.0226	<0.001
Digestive tract infection	0.0147 ± 0.0632	0.0025 ± 0.0226	0.006
Other sites of infection	0.0251 ± 0.1855	0.0025 ± 0.0226	0.006
Total infection	0.1388 ± 0.2219	0.0637 ± 0.1230	<0.001

*URTI, upper respiratory tract infection*.

### Association of Baseline Characteristics and Infection Incidence Before and During the COVID-19 Pandemic

A correlation analysis was conducted to reveal the association between baseline characteristics and the incidence of infectious diseases. Before the pandemic, catheter-related infections were negatively related to the period of hemodialysis due to the fact that catheters were mainly used in the initial stages of hemodialysis. Digestive tract infections were associated with diabetes, higher diastolic pressures, and shorter periods of hemodialysis. URTI was associated with longer periods of hemodialysis. Furthermore, pneumonia was associated with age, cardiovascular diseases, serum phosphorus levels, and a need for higher erythropoietin dosages. Thus, respiratory infections were positively associated with age and the need for higher erythropoietin dosages. The total infection incidence was associated with age, the presence of obstructive kidney disease, and the need for higher erythropoietin dosages (Supplementary Table 1).

During the pandemic, catheter-related infections were related to brain vascular diseases. Digestive tract infections were associated with cardiovascular diseases. UTRI positively correlated with BMI and the length of hemodialysis. And respiratory infections and the total infection incidence positively correlated with serum calcium levels and the periods of hemodialysis. The risk factors for infection differed between the duration of the pandemic and before it (Supplementary Table 2).

## Discussion

Preventive measures against infections, especially respiratory and digestive tract infections, are limited in patients undergoing hemodialysis (15). Hand hygiene was the most acknowledged method for prevention against infections (16–18); however, the compliance rates were disappointing among HCWs, and much less among patients undergoing hemodialysis and the general population (19, 20). Thus, hand hygiene is recommended for HCWs in order to prevent the transmission of the virus *via* their hands (3, 21). Surgical masks were used mainly by HCWs in East Asia (3, 12); however, their efficacy has been controversial. Some studies report that the usage of surgical masks decreased the incidence of influenza-like illnesses in pilgrim crowds (22). However, without hand hygiene, it was not effective in preventing influenza among HCWs during the period of the epidemic (23). The effects of vaccines against influenza and pneumovax were also limited (3, 24). For patients undergoing hemodialysis, hand hygiene, wearing masks in the presence of respiratory symptoms, and vaccinations have been recommended (3, 5); however, this was not mandatory before the COVID-19 pandemic. In the present study, these measures were recommended for patients undergoing hemodialysis before the COVID-19 pandemic. However, the compliance rates were disappointing. During the pandemic, the compliance rates increased critically owing to mandatory policies. Consequently, the incidences of respiratory, catheter-related, and digestive tract infections all decreased during the pandemic. These results indicated that mitigation measures against COVID-19 reduced the incidences of respiratory, catheter-related, and digestive tract infections among patients undergoing hemodialysis.

The supplemented prevention regulations on the behaviors of HCWs in the hemodialysis unit during the pandemic were mainly screening patients suspected to have COVID-19, mandatory mask-wearing, and reinforced hand hygiene (5). Thus, the compliance with hand hygiene increased during the pandemic, especially in nurses and other employees. The increase in hand sanitizer consumption during the pandemic supported these results. Previous studies also support the practice of hand hygiene in HCWs (3, 16, 17). These regulations might minimize the transmission of pathogens through the hands of HCWs.

The COVID-19 prevention regulations on the behaviors of patients and caregivers changed their behaviors critically (7, 12). The mask-wearing rate increased from 1.5 to 100.0%, and the compliance with hand hygiene increased, as indicated by the fundamental increase in hand sanitizer consumption during the pandemic. Mask-wearing might decrease facial touching behaviors and prevent environmental surface contamination in patients with respiratory infections. In China, an emergency shutdown was conducted on January 23, 2020, after which the reopening of the society starting gradually from February 10 (7, 25). This might have influenced the incidences of infectious diseases. However, the efficacy of the emergency shutdown in curbing the transmission of COVID-19 is controversial (26). Moreover, the patients undergoing hemodialysis were not as socially active as normal people, which also limited the efficacy of the emergency shutdown on the prevention of infectious diseases.

The prevention efficacy of the mitigation measures against COVID-19 on infectious disease episodes might lie in reducing the contact transmission of infectious pathogens. Catheter-related infections, digestive tract infections, URTIs, and pneumonia all decreased during the COVID-19 pandemic. They shared the same transmission route, that is, contact transmission (3). Thus, the most effective measures should be increased hand hygiene and mask-wearing in symptomatic patients with respiratory infections. Mask-wearing might cause inconvenience to those without respiratory infections; however, studies have indicated that mask-wearing might reduce face-touching behaviors, which might prevent respiratory infections such as influenza and COVID-19 (12, 14, 27). Thus, mandatory mask-wearing regulations might reduce the healthcare costs for patients and caregivers in the hemodialysis unit.

The practices for infection prevention among patients undergoing hemodialysis were mainly based on the evidence from general populations rather than those uniquely for patients undergoing hemodialysis (1, 3, 6). Catheter-related infections were hemodialysis-specific and most studied (17, 28, 29). Previous studies found that catheter-related infections could be reduced from 2.04 per 100 patient-months preintervention to 0.75 per 100 patient-months (*p* = 0.03) after employing the collaborative interventions and to 0.24 (*p* < 0.01) per 100 patient-months after augmenting the collaborative interventions with positive deviance (28). In the present study, catheter-related infections decreased from a mean of 0.93 per 100 patients per month to 0.25 per 100 patients per month (*p* = 0.003). The overall incidence rate of pneumonia for the study period was 5.42 events per 100 patient-months before the COVID-19 pandemic, which was higher than that in previous reports (4). This might be due to lower BMIs and hemoglobin levels in the patients enrolled in this study compared with those in the patients in previous reports (4, 30). During the pandemic, the incidence rate of pneumonia decreased to 2.04 events per 100 patient-months. Guidelines and specific methods cannot cover all the different situations that arise during the care continuum and can paradoxically result in an increased spread of hospital infections (15). It is difficult to directly compare the efficacies of different infection prevention methods. Thus, the current study indicated the association between mandatory mask-wearing and reinforced hand hygiene education and decreased infection episodes in patients undergoing hemodialysis.

### Limitation

This study was conducted in a single center. Thus, the results need to be tested in multiple centers and multiple regions with different cultures, COVID-19 incidences, and public health policies (15). The preventive effects of the mitigation of COVID-19 on infectious episodes among patients undergoing hemodialysis need confirmation by clinical trials and other studies worldwide. The compliance rate of mask-wearing regulations might be easily improved in the hemodialysis units in East Asia (12). However, the situation might differ in regions with low mask-wearing compliance. In addition, reinforced hand hygiene education needs more technical and friendly support in order to increase the compliance rate based on local cultures and regulations.

Above all, the present study indicated the association between mandatory mask-wearing and reinforced hand hygiene education and decreased respiratory, catheter-related, and digestive tract infection episodes in the hemodialysis unit.

## Data Availability Statement

The original contributions generated for the study are included in the article/Supplementary Material, further inquiries can be directed to the corresponding authors.

## Ethics Statement

The studies involving human participants were reviewed and approved by the Clinical Ethics Review Board of the Third Affiliated Hospital of Guangzhou Medical University. The patients/participants provided their written informed consent to participate in this study.

## Author Contributions

XL, JC, and Y-FX had full access to all of the data in the study and take responsibility for the integrity of the data and the accuracy of the data analysis and supervision. J-JQ, Y-FX, J-HR, Y-JC, Y-FG, Y-QJ, JC, XL: concept and design, acquisition, analysis, or interpretation of data, drafting of the manuscript, and Critical revision of the manuscript for important intellectual content. XL, J-HR, and Y-JC: statistical analysis. XL and Y-FX: obtained funding. J-JQ, J-HR, Y-FG, Y-QJ: administrative, technical, or material support. All authors contributed to the article and approved the submitted version.

## Conflict of Interest

The authors declare that the research was conducted in the absence of any commercial or financial relationships that could be construed as a potential conflict of interest.

## Abbreviations

ESRD: end-stage renal disease
COVID-19: coronavirus disease 2019
HCWs: health care workers
BMI: body mass index
PTH: parathyroid hormone
URTI: upper respiratory tract infection
SMD: standardized mean difference.

## References

[1] Hess S Bren V. Essential components of an infection prevention program for outpatient hemodialysis centers. Semin Dial. (2013) 26:384–98. 10.1111/sdi.12102 23808676

[2] Xing YF Cai RM Lin Q Ye QJ Ren JH Yin LH. Expansion of polymorphonuclear myeloid-derived suppressor cells in patients with end-stage renal disease may lead to infectious complications. Kidney Int. (2017) 91:1236–42. 10.1016/j.kint.2016.12.015 28215666

[3] Karkar A Bouhaha BM Dammang ML. Infection control in hemodialysis units: a quick access to essential elements. Saudi J Kidney Dis Transpl. (2014) 25:496–519. 10.4103/1319-2442.132150 24821145

[4] Sibbel S Sato R Hunt A Turenne W Brunelli SM. The clinical and economic burden of pneumonia in patients enrolled in medicare receiving dialysis: a retrospective, observational cohort study. BMC Nephrol. (2016) 17:199. 10.1186/s12882-016-0412-6 27955633 PMC5153919

[5] Basile C Combe C Pizzarelli F Covic A Davenport A Kanbay M. Recommendations for the prevention, mitigation and containment of the emerging SARS-CoV-2 (COVID-19) pandemic in haemodialysis centres. Nephrol Dial Transplant. (2020) 35:737–41. 10.1093/ndt/gfaa069 32196116 PMC7184437

[6] Akinbodewa AA Adejumo OA. Awareness and practice of vaccination of chronic hemodialysis patients by specialist nephrology practitioners in nigeria: a cross-sectional survey. J Epidemiol Glob Health. (2019) 9:204–9. 10.2991/jegh.k.190518.001 31529939 PMC7310821

[7] Health Committee of Guangdong Province. Guangdong province has decided to launch a first-level response to a major public health emergency, People's Government of Guangdong Province, Guangzhou (2020).

[8] Pan X Chen D Xia Y Wu X Li T Ou X. Asymptomatic cases in a family cluster with SARS-CoV-2 infection. Lancet Infect Dis. (2020) 20:410–1. 10.1016/S1473-3099(20)30114-6 32087116 PMC7158985

[9] Wang R Liao C He H Hu C Wei Z Hong Z. COVID-19 in hemodialysis patients: a report of 5 cases. Am J Kidney Dis. (2020) 76:141–3. 10.1053/j.ajkd.2020.03.009 32240718 PMC7118604

[10] Xiong F Tang H Liu L Tu C Tian JB Lei CT. Clinical characteristics of and medical interventions for COVID-19 in hemodialysis patients in Wuhan, China. J Am Soc Nephrol. (2020) 31:1387–97. 10.1681/ASN.2020030354 32385130 PMC7350995

[11] Guan WJ Liang WH Zhao Y Liang HR Chen ZS Li YM. Comorbidity and its impact on 1590 patients with COVID-19 in China: a nationwide analysis. Eur Respir J. (2020) 55:2000547. 10.1183/13993003.00547-2020 32217650 PMC7098485

[12] Chen Y-J Qin G Chen J Xu J-L Feng D-Y Wu X-Y. Comparison of face-touching behaviors before and during the coronavirus Disease 2019 pandemic. JAMA Netw Open. (2020) 3:e2016924. 10.1001/jamanetworkopen.2020.16924 32725247 PMC12124488

[13] Zhang R Li Y Zhang AL Wang Y Molina MJ. Identifying airborne transmission as the dominant route for the spread of COVID-19. Proc Natl Acad Sci USA. (2020) 117:14857–63. 10.1073/pnas.2009637117 32527856 PMC7334447

[14] Chu DK Akl EA Duda S Solo K Yaacoub S Schunemann HJ. Physical distancing, face masks, and eye protection to prevent person-to-person transmission of SARS-CoV-2 and COVID-19: a systematic review and meta-analysis. Lancet. (2020) 395:1973–87. 10.1016/j.jvs.2020.07.040 32497510 PMC7263814

[15] Gesser-Edelsburg A Cohen R Halavi AM Zemach M van Heerden PV Sviri S. Beyond the hospital infection control guidelines: a qualitative study using positive deviance to characterize gray areas and to achieve efficacy and clarity in the prevention of healthcare-associated infections. Antimicrob Resist Infect Control. (2018) 7:124. 10.1186/s13756-018-0418-x 30386593 PMC6201509

[16] Lyons PG Kollef MH. Prevention of hospital-acquired pneumonia. Curr Opin Crit Care. (2018) 24:370–8. 10.1097/MCC.0000000000000523 30015635

[17] Hsu V. Prevention of health care-associated infections. Am Fam Physician. (2014) 90:377–82. 25251230

[18] Sim SW Moey KS Tan NC. The use of facemasks to prevent respiratory infection: a literature review in the context of the health belief model. Singapore Med J. (2014) 55:160–7. 10.11622/smedj.2014037 24664384 PMC4293989

[19] Gould DJ Moralejo D Drey N Chudleigh JH Taljaard M. Interventions to improve hand hygiene compliance in patient care. Cochrane Database Syst Rev. (2017) 9:CD005186. 10.1002/14651858.CD005186.pub4 28862335 PMC6483670

[20] McLaws ML Kwok YLA. Hand hygiene compliance rates: fact or fiction? Am J Infect Control. (2018) 46:876–80. 10.1016/j.ajic.2018.03.030 29778435

[21] Kou M Hwang V Ramkellawan N. Bronchiolitis: from practice guideline to clinical practice. Emerg Med Clin North Am. (2018) 36:275–86. 10.1016/j.emc.2017.12.006 29622322

[22] Barasheed O Almasri N Badahdah AM Heron L Taylor J McPhee K. Pilot randomised controlled trial to test effectiveness of facemasks in preventing influenza-like illness transmission among Australian Hajj pilgrims in 2011. Infect Disord Drug Targets. (2014) 14:110–6. 10.2174/1871526514666141021112855 25336079

[23] Chughtai AA Seale H MacIntyre CR. Availability, consistency and evidence-base of policies and guidelines on the use of mask and respirator to protect hospital health care workers: a global analysis. BMC Res Notes. (2013) 6:216. 10.1186/1756-0500-6-216 23725338 PMC3693993

[24] Van Buynder PG Konrad S Kersteins F Preston E Brown PD Keen D. Healthcare worker influenza immunization vaccinate or mask policy: strategies for cost effective implementation and subsequent reductions in staff absenteeism due to illness. Vaccine. (2015) 33:1625–8. 10.1016/j.vaccine.2015.01.048 25678243

[25] People's Government of Guangdong Province. Notice of the People's Government of Guangdong Province on the resumption of business and the opening of schools, People's Government of Guangdong Province. Guangzhou: People's Government of Guangdong Province (2020).

[26] Wang G Zhang Y Zhao J Zhang J Jiang F. Mitigate the effects of home confinement on children during the COVID-19 outbreak. Lancet. (2020) 395:945–47. 10.1016/S0140-6736(20)30547-X 32145186 PMC7124694

[27] Long Y Hu T Liu L Chen R Guo Q Yang L. Effectiveness of N95 respirators versus surgical masks against influenza: a systematic review and meta-analysis. J Evid Based Med. (2020) 10:779. 10.1111/jebm.12381 32167245 PMC7228345

[28] Lindberg C Downham G Buscell P Jones E Peterson P Krebs V. Embracing collaboration: a novel strategy for reducing bloodstream infections in outpatient hemodialysis centers. Am J Infect Control. (2013) 41:513–9. 10.1016/j.ajic.2012.07.015 23219669

[29] Golestaneh L Mokrzycki MH. Prevention of hemodialysis catheter infections: ointments, dressings, locks, and catheter hub devices. Hemodial Int. (2018) 22:S75–82. 10.1111/hdi.12703 30411464

[30] Chen J Qin X Li Y Yang Y Yang S Lu Y. Comparison of three nutritional screening tools for predicting mortality in maintenance hemodialysis patients. Nutrition. (2019) 67–68:110532. 10.1016/j.nut.2019.06.013 31445314

